# Alveolar Recruitment for ARDS Trial: preliminary results

**DOI:** 10.1186/cc12047

**Published:** 2013-03-19

**Authors:** AB Cavalcanti, EA Suzumura, M Abreu, GF Ribeiro, A Kodama, F Moreira, HP Guimarães, E Romano, MB Amato, O Berwanger, CR Carvalho, ART Investigators

**Affiliations:** 1 Hospital do Coração - HCor, São Paulo, Brazil; 2 Universidade de São Paulo, Brazil

## Introduction

The aim of the ongoing Alveolar Recruitment for ARDS Trial (ART) is to evaluate whether a maximum stepwise alveolar recruitment maneuver (MSARM) followed by ventilation at optimal PEEP may decrease 28-day mortality in patients with moderate to severe ARDS compared with ventilation with the ARDSNet strategy (NCT01374022). Here we report the results of a planned *a priori *preliminary analysis involving 100 patients to assess feasibility, physiologic variables and safety outcomes.

## Methods

ART is an event-driven multicenter randomized controlled trial planned to last until 520 deaths within 28 days are observed. Patients assigned to the experimental group receive a MSARM achieving PEEP of 45 cmH_2_O and plateau pressure of 60 cmH_2_O plus PEEP titrated according to the static compliance of the respiratory system (ART strategy). The target tidal volume is 4 to 6 ml/kg predicted body weight (PBW) and plateau pressure ≤30 cmH_2_O in both groups.

## Results

We randomized 101 patients between November 2011 and October 2012 in 51 centers. Considering 124 active sites in July 2013 and the current recruitment rate, we will finish enrollment by November 2014. MSARM was complete in 34/47 (72%) of patients allocated to the ART group. The main reason for MSARM interruption was hypotension (8/9 (90%)). Mean titrated PEEP in the ART group was 16.1 ± 3.5 versus 12.9 ± 3.4 cmH_2_O in ARDSNet (*P <*0.001). One hour after randomization, tidal volume was similar between the ART and ARDSNet groups (5.1 ± 0.8 vs. 5.3 ± 0.7 ml/kg PBW, respectively; *P *= 0.08). Mean values remained below 6 ml/kg PBW up to day 3. Few patients had plateau pressure >30 cmH_2_O 1 hour after randomization in the ART and ARDSNet groups (3/43 (7.0%) vs. 4/47 (8.5%), respectively; *P *= 1.00) and on subsequent days. PaO_2_/FiO_2 _was significant higher in the ART group (179.5 ± 84.5 vs. 143.3 ± 46.8, *P *= 0.01) and increased over time up to 7 days after randomization (272.3 ± 136.5 vs. 192.6 ± 72.3, *P *= 0.003). The ART strategy did not increase the risk of barotrauma (relative risk (RR) = 0.78, 95% CI = 0.19 to 3.30) in the first 7 days after randomization or the need to initiate or increase vasopressors or mean arterial pressure <65 mmHg (RR = 1.14, 95% CI = 0.65 to 2.02, *P *= 0.67) 1 hour after randomization. However, the ART strategy increased the risk for severe acidosis (pH <7.10) 1 hour after randomization (RR = 3.20, 95% CI = 1.12 to 9.20, *P *= 0.03).

## Conclusion

ART is feasible. The incidence of adverse events was similar between groups except for severe acidosis 1 hour after randomization. Hence we adjusted the study protocol, increasing the respiratory rate (from 10 to 15/minute) during MSARM.

